# The role of heat shock proteins in placental ischemic disease: A narrative review of the current literature

**DOI:** 10.1002/ijgo.70039

**Published:** 2025-03-03

**Authors:** Athina A. Samara, Anastasios Lafioniatis, Maria Ioannou, Sofia Tsiapakidou, Angeliki Gerede, Eleftherios Anastasakis, Alexandros Daponte, Sotirios Sotiriou

**Affiliations:** ^1^ Department of Embryology, Faculty of Medicine University of Thessaly Larissa Greece; ^2^ Department of Obstetrics and Gynecology University Hospital of Larissa Larissa Greece; ^3^ Klinikum am Gesundbrunnen SLK‐Kliniken Heilbronn GmbH Heilbronn Germany; ^4^ Department of Pathology, Faculty of Medicine University of Thessaly Larissa Greece; ^5^ 1st Department of Obstetrics and Gynecology Aristotle University of Thessaloniki Greece Thessaloniki Greece; ^6^ Unit of Maternal‐Fetal‐Medicine, Department of Obstetrics and Gynecology, Medical School Democritus University of Thrake Alexandroupolis Greece; ^7^ Iaso Maternity Hospital Athens Greece

**Keywords:** FGR, heat shock, placenta, pre‐eclampsia

## Abstract

Pre‐eclampsia, placental abruption, and fetal growth restriction (FGR) are collectively referred to as placental ischemic disease (PID). Heat shock proteins (HSPs), originally considered as a response to the heat shock, have a central role in regulating the cellular functions by quality controlling the newly synthesized proteins. The aim of the present review is to investigate the expression of the HSPs in PID and their potential role as biomarkers, based on the available data in the literature. A considerable amount of research has been conducted in order to determine the significance of HSPs in placental pathology and insufficiency, using both immunochemistry and circulating mRNA approaches. HSPs seem to be promising biomarkers that could be used for screening and monitoring the cellular stress of the placenta and its dysfunction. Yet, in order to be able to reach more solid evidence and draw a safer conclusion regarding their utility in clinical practice there is still a long way to go and further well‐designed greater scale studies are required.

## 1 INTRODUCTION

Pre‐eclampsia, placental abruption, and fetal growth restriction (FGR) are collectively referred to as placental ischemic disease (PID). This classification arises from their common features of uteroplacental malperfusion, chronic hypoxia, and placental ischemia. These conditions result from inadequate trophoblast invasion and incomplete remodeling of the spiral arteries during placentation.^1^, ^2^, ^3^ At approximately 8–10 weeks of gestation, the maternal vessels undergo remodeling, thereby eliminating cytotrophoblast plugging and permitting perfusion of the intervillous space. This augmented perfusion not only enhances the delivery of nutrients and oxygen to the embryo but also precipitates two particularly significant outcomes. First, the increased oxygen delivery induces oxidative stress, necessitating buffering mechanisms. Second, the elevated partial pressure of oxygen (pO_2_) facilitates the conversion of cytotrophoblasts into an invasive phenotype.^3^

In non‐pregnant women, spiral arteries represent the terminal branches of the uterine arteries, responsible for supplying blood to the endometrium. During a normal pregnancy, between 10 and 20 weeks of gestation, these vessels undergo substantial physiological alterations.^4^ The most prominent change is a significant increase in the terminal luminal diameter, expanding by 5‐ to 10‐fold. Additionally, the structure of the vessel wall is modified as the muscular and elastic components are eliminated, transforming the vessel into a flaccid, dilated tube. This remodeling extends into the inner third of the myometrium.^5^ The depth of this vascular invasion is particularly important. Near the interface of the uterine mucosal lining (endometrium in non‐pregnant women and decidua during pregnancy), there exists a segment of vascular smooth muscle that acts as a functional sphincter. During menstruation, this segment constricts to terminate bleeding that occurs when the spiral artery is disrupted by endometrial shedding. However, the physiological remodeling associated with pregnancy results in the loss of this sphincter mechanism.^5^ Combined with the loss of smooth muscle in the spiral arteries within the decidua and the inner third of the myometrium, this portion of the vessel loses its ability to contract in response to humoral or neural stimuli.^6^ Additionally, there is an increase in the formation of vascular shunts between arteries and veins beneath the placenta, although the significance of this change remains unclear.^3^ In a subset of pregnant women, these physiological modifications fail to transpire. The terminal segments of the vessels do not undergo the expected dilation, smooth muscle persists within the vessel walls, and the extent of vascular remodeling does not extend beyond the decidual layer and consequently, the “functional sphincter” is maintained.^3^ These findings are very similar in pregnancies with IPD.

Pre‐eclampsia and FGR are two well recognized contributors to maternal and neonatal morbidity and mortality. Although a lot of research has been done studying these conditions, current diagnostic criteria often fail to set the correct diagnosis which has great consequences.^7^ The advent of molecular techniques has brought ton of knowledge concerning potential biomarkers which could be utilized for diagnosis and follow up of patients. Global warming is also a global phenomenon that is known to affect the reproduction of European cattle and heat shock is suspected to be the principal factor affecting their reproductive function.^8^ This of course creates new possibilities for the research of fertility and reproductive physiology of humans. Heat shock proteins (HSPs) are some of those molecules and their crucial role in regulating protein trafficking and cellular homeostasis renders them as potential candidates.

The aim of the present review was to investigate the expression of the HSPs in PID and their potential role as biomarkers, based on the available data in the literature.

## 2 FETAL GROWTH RESTRICTION (FGR)

Fetal growth restriction (FGR), also known as intrauterine growth restriction (IUGR), is a well‐known recognized risk factor for a wide range of negative obstetric and neonatal outcomes such as still birth and preterm birth. It is also linked with a variety of conditions that develop later in life.^9^ It is therefore of great importance to timely recognize fetuses at risk of FGR in order to monitor these pregnancies more closely and also to properly intervene if possible.^10^ This way the ultimate goal of prenatal medicine which is reducing maternal and neonatal mortality and morbidity can be achieved.

Despite its significance, FGR lacks a widely accepted definition. Therefore, the prevalence of FGR varies in studies depending on the definition that had been used.^11^ The most commonly applied definition takes into consideration the estimated fetal weight (EFW) or the abdominal circumference (AC). In order to calculate the EFW most clinicians use the so called Hadlock formula.^12^, ^13^ If those values fall under the 10th percentile the diagnosis of FGR is established, whereas if they are less than the third percentile FGR is characterized as severe. This definition is problematic as it fails to correctly identify fetuses who do not achieve their growth potential and yet their EFW or AC never fall below the 10th percentile while at the same time it classifies as FGR fetuses which are constitutionally small.^14^, ^15^, ^16^

The frequency of erroneous calculations of EFW and the actual birth weight was up to 14% among various studies suggesting that this limitation is also contributing to the misdiagnosis of FGR.^17^, ^18^ FGR is further characterized as early or late onset if it manifests before or after 32 weeks of gestation, respectively, and asymmetric when only the AC is less than the cutoff value or symmetric when the head, body and length of the fetus are equally affected. Another term that generally causes confusion among clinicians is that of small for gestational age (SGA) fetuses which refers to all newborns with a birth weight of less than the 10th percentile for their gestational age.^11^

FGR is thought to stem from a variety of factors namely maternal, fetal and placental. Although the pathophysiologic process in every case is different the final pathway seems to be common: a pathologic uteroplacental circulation and the subsequent suboptimal fetal nutrition.^1^ Placental insufficiency is recognized as an important contributor to the pathogenesis of FGR.^1^, ^3^ Therefore, the histologic examination of the placenta has been for a long time the studying objective of many researchers that try to better understand the sequence of events that ultimately lead to the development of FGR.^19^

The recent advances in molecular biology have brought to light a great number of molecules and intertwining pathways that allow us to better understand the pathologic processes of cellular stress. One of the key molecules that are known for their protective role against cellular stress and damage are the so‐called HSPs more colloquially known as molecular chaperones.^20^ These proteins have been thoroughly studied regarding their role in FGR. Although their contribution is not fully understood there is a great amount of evidence that support their utility in understanding the pathophysiologic aspects and in screening and more accurately diagnosing FGR.^20^

## 3 PRE‐ECLAMPSIA

Pre‐eclampsia is another clinical entity sharing common pathogenetic pathways with PID that is known for its contribution to both maternal and neonatal mortality and morbidity. Pre‐eclampsia is defined as elevated blood pressure ≥140/90 mmHg accompanied with a proteinuria of more than 300 mg/24 h occurring after the 20th week of pregnancy.^21^ As a multisystem disorder pre‐eclampsia can affect almost any system including the cardiovascular system, the central nervous system, the liver, hemostasis and the kidneys. It is estimated that about 4.6% of pregnancies may develop pre‐eclampsia whereas a small amount of pre‐eclamptic patients may demonstrate a particular subtype called hemolysis, elevated liver enzymes and low platelets (HELLP) syndrome.^22^ Although we have still not achieved complete understanding of the complex mechanisms that lead to its manifestation, the clinical and laboratory findings of pre‐eclampsia seem to arise from a systemic vascular dysfunction and activation leading to vasospasm.^23^ During the last decades, research has linked pre‐eclampsia with many late maternal complications including increased risk for cardiovascular disease and cognitive impairment.^24^

As known, risk factors for the development of pre‐eclampsia are nulliparity, history of pre‐eclampsia in a previous pregnancy, diabetes mellitus, multifetal pregnancy, pre‐existing renal and autoimmune disorders.^25^ Over the years there has been a great effort in order to identify potential sonographic and molecular markers in order to correctly identify the women at risk and properly intervene in time. Uterine artery Doppler has for a long time been the main screening method.^26^, ^27^ The progress achieved in our understanding the molecular event leading to pre‐eclampsia led to the introduction of sFLT 1, an antiangiogenic factor, and PlGF a proangiogenic factor and their ratio.^28^, ^29^, ^30^, ^31^ Yet, there is to this day a lack of uniformly accepted markers that can be implemented in order to be able to classify pre‐eclampsia as mild or heavy and on the other hand there are no markers that can be used in order to assess the response to therapy. Acting as buffers against cellular stress, struggling to maintain proteostasis, HSP detection could prove to be a useful tool in studying the placental insufficiency which is related to pre‐eclampsia.

## 4 HEAT SHOCK PROTEINS (HSPs)

In 1962 Ritossa reported that treating Drosophila chromosomal squashes at 37°C induced a different puffing pattern than the expected.^32^ Fifteen years later, Lewis and Tissiéres in their respected studies investigated this phenomenon on the molecular level, reported that from these puffs mRNAs of proteins were being synthesized in response to the heat shock.^33^, ^34^ Based on the original observations, these proteins were named HSPs; however, it was later found that they were also produced in many stress circumstances including as a response to other harmful stimuli such as radiation and arsenic exposure.

According to their molecular weight in Dalton (kDa), measured using electrophoresis, HSPs were classified into six groups namely large HSPs, HSP90, HSP70, HSP60, HSP40 and small HSPs.^35^ These molecules participate in one of the most critical for the survival of the cell function which is that of proteostasis. Their subcellular localization and function of each protein of this group vary widely. HSP can be located in the nucleus, the endoplasmic reticulum and the cytosol of the mitochondria. Their catalytic actions have been described as holdases catalyzing the non‐covalent folding of proteins in an adenosine triphosphate (ATP) dependent manner. HSP sequestrases bind to specific proteins and isolate them in specific protein complexes separating them from their binding partners not enabling them to display their biologic roles, aggregases promote the aggregation of proteins and disaggregases dissolve and clear already formed protein aggregates. HSPs interact with numerous cochaperones that regulate their activity.^36^

In contrast to proteins with up to 100 amino acids, where folding can be achieved spontaneously, proteins with more than 100 amino acids require the support of an intricate network of chaperones.^37^ In eukaryotes the newly synthesized polypeptides associate with nascent chain associated complex (NAC) and ribosome associated complex (RAC) which then deliver them to HSP70/HSP40 which functions as an intermediate step for further protein folding processes.^38^ HSP70 recognize hydrophobic patterns within the nascent polypeptide thus promoting the folding of intracellular proteins once they have been synthesized. In subsequent steps in this multilayered process, TRiC chaperone facilitates further folding of proteins by recognizing folding intermediates that are formed by HSP70 whereas HSP90 recognizes its substrates based on their physicochemical and conformational characteristics.^39^ Apart from folding newly synthesized polypeptides, partially misfolded or unfolded proteins are recognized by HSPs and based on their condition they are triaged to be either rescued by restoring the proper folding pattern or are targeted to be degraded by either ubiquitination and subsequent proteosomic proteolysis or by means of autophagy or lysosomal pathways.^35^

HSPs are highly conserved among prokaryotes and eukaryotes. Many of them execute their housekeeping function by tightly regulating the trafficking, folding, degradation and prevention of aggregation of newly synthesized proteins. By regulating the cellular proteome, HSPs influence also greatly other cellular functions including signal transduction and cell survival. It should be noted that through their function most chaperones are shown to have prosurvival roles by stabilizing antiapoptotic protein complexes, whereas HSP60 in particular has been shown to exhibit both anti‐ and proapoptotic properties.^35^ Moreover, apart from their house keeping function, many HSPs are activated and their expression is upregulated as a cellular response to stress. Many of these HSPs are regulated by so called heat shock factors (HSF). These proteins bind to HSPs non‐covalently and once the cells are exposed to harmful stimuli detach from them, translocate to the nucleus and activate the transcription of HSPs.^35^

Many human diseases have been linked to HSPs. This is not a surprise if one takes into account their central role in regulating the cellular functions by quality controlling the newly synthesized proteins. In this context their role in placental dysfunction has attracted the attention of many researchers. A considerable amount of research has been conducted in order to determine the significance of HSPs in placental pathology and insufficiency which are widely regarded as an important contributing factor to the pathogenesis of FGR and pre‐eclampsia.^20^

## 5 PLACENTAL PATHOLOGY AND HSPs

The placenta is a very important organ that functions in a multifaceted way throughout pregnancy. During intrauterine development, gas exchange is performed exclusively by the placenta, since the fetal lungs are filled with fluid and cannot accommodate this function. Moreover, the placenta facilitates the absorption of nutrients and the excretion of metabolic byproducts and at the same time produces a variety of both protein and steroid hormones. Last but not least, it is theorized to participate in intricate immunologic interactions with the decidua.^40^ It has been long hypothesized that the dysfunction of the placenta can contribute to the pathogenesis of FGR.

In order to understand the mechanisms of placental insufficiency, scientists in the past used to apply histopathologic terms. The term syncytial knot count refers to aggregates of cellular nuclei whereas in the normal syncytium the nuclei are expected to be evenly distributed in the cytoplasm. Choriangiosis is the presence of more than 10 capillaries per villus whereas in avascular villi a complete shrinkage of capillaries is observed. Fibrin deposition is when the villi are circumferentially covered with fibrin. Placental infarction is a collection of villi which have undergone ischemic necrosis as a result of maternal hypoperfusion in certain areas of the placental bed. Thrombosis refers to the complete occlusion of the capillary lumens by thrombi.^36^

The role of HSP in placental pathology and its relationship with inflammatory processes caught the attention of researchers during the last decades. Molvarec et al.,^41^ reported that high serum levels of HSP70 were associated with systemic inflammation and oxidative stress in pregnancies complicated by pre‐eclampsia. This inflammation process contributes to endothelial dysfunction, that plays a critical role in placental insufficiency. The association between HSP70 and proinflammatory cytokines of monocytes exposed to Toll‐like receptors (TLR) agonists suggest that HSP70 can negatively regulate inflammatory responses in the placenta.^42^ Moreover, Fukushima et al.^43^ measured HSP70 serum levels particularly high in treatment‐resistant preterm delivery cases and concluded that HSP70 may prove to be a useful marker for evaluating the curative effects of treatment for preterm delivery. These findings underline the key role of HSPs in negative feedback with inflammatory pathways activated in the placentas in stressed induced situations.

Several different pathologic conditions during pregnancy can affect placenta. Sharma and Shukla^44^ reported that high levels of HSPs in malaria‐infected placentas are important to maintain the normal placental architecture and physiology and suggested that treatment should be focused on the increase of HSP levels in these placentas. At the same time, Chaiworapongsa et al.^45^ investigated HSP70 amniotic fluid concentrations in histologic chorioamnionitis. HSP70 values were statistically significant higher in amniotic fluids of pregnancies complicated with chorioamnionitis, underlining the role of HSPs in intrauterine infections.^45^ In summary, HSPs have a protective role in the homeostasis of pregnancy in many inflammatory pathologic conditions.

## 6 HEAT SHOCK PROTEINS IN PLACENTAL ISCHEMIC DISEASE: MECHANISMS, FETAL DEVELOPMENT IMPACT, AND THERAPEUTIC POTENTIAL FOR ENHANCED PREGNANCY OUTCOMES

Many researchers over the years have conducted studies in order to illuminate the potential role of various molecules associated with cellular stress in various histopathologic changes in the placentas of FGR fetuses. Wataba et al.,^36^ examined the placentas of FGR fetuses and reported that in placentas with thrombi, excessive syncytial knot and avascular villi all HSPs were significantly higher expressed in comparison with the healthy controls. Additionally, in infarction zones HSP expression was lower whereas no difference was observed in fibrin deposition or choriangiosis.^36^ Ziegert et al.,^46^ in another study of 12 preterm deliveries anti‐HSP70 antibodies and anti‐HSP60 antibodies were detected and the authors concluded that the formation of these anti‐HSP antibodies could contribute to the induction of preterm delivery. Furthermore, a study by Barut et al.^47^ included 135 placental villous specimens from healthy, pre‐eclamptic and FGR gestations, and a clearly higher expression of HSP70 and endothelial NO synthase (eNOS) were detected using immunohistochemistry. Moreover, Chen et al.^48^ demonstrated that disruption of the HSP90‐eNOS interaction and of the phosphatidylinositol 3 (PI3)‐kinase (PI3K‐Akt) pathway blocks Ang‐1‐stimulated endothelial cell migration and formation of complex capillary networks. HSPs play an important role in the complex molecular mechanisms of angiogenesis and they may provide the foundation for future therapies for the prevention and reversal of artery diseases.^48^ In contrast, Hnat et al.^49^ in a study of five healthy normotensive pregnancy placental tissues, five hypertensive, five IUGR and four concurrently IUGR and hypertensive placentas, no difference was observed in the expression of HSP70 and HNE and the authors concluded that these two biomarkers should not be used in the assessment of oxidative stress in placentas.

Moreover, Álvarez‐Cabrera et al.^50^ measured extracellular HSP 60 and 70 values in pre‐eclampsia‐complicated pregnancies and reported a positive correlation between them and markers indicating hepatic dysfunction including uric acid, lactate dehydrogenase (LDH), glutamic oxaloacetic transaminase (GOT) glutamic pyruvic transaminase (GPT), and inflammatory IL‐1β and TNFα response. However, according to Sisti et al.^51^ there is not necessarily a correlation between intra‐ and extracellular levels of HSP70.

Liu et al.^52^ also associated HSP70 in the pathogenesis of PID and suggested that it may provide an explanation for the endothelial cell activation and proinflammatory response of the disease. Higher expression of intracellular HSP70 provided a protective role against oxidative stress and extracellular HSP70 may initiate an inflammatory response in PID.^53^ However, Molvarec et al.^54^ reported different levels of HSP70 in early and late‐onset pre‐eclampsia, adding evidence in the different pathophysiologic etiology between the disease, highlighting the greater severity of the early‐onset disease.

In summary, several studies now underline the role of HSPs in the PID, not only as molecular chapons but also as potential biomarkers and therapeutic targets to gestational outcomes. Particularly HSP70 and HSP90 activate protective mechanisms that respond to cell stress induced by the placental ischemia, affecting fetal development by modulating apoptosis and angiogenesis. To date, HSP90 and HSP70 have been extensively investigated in terms of drug discovery and they have received the most interest in the past two decades. HSP90 inhibitors are the most advanced among HSPs, and more than 20 inhibitors have undergone clinical trials, some of which are limited by adverse toxicities during several clinical evaluation and none has yet been approved by the FDA.^35^

## 7 CIRCULATING HSP MARKERS AS A POTENTIAL SCREENING TOOL

The lack of a generally accepted definition and the limitations posed by ultrasonography screening in diagnosing fetuses at risk of developing PID, dictates the need for new prognostic and diagnostic biomarkers. As mentioned above, current literature indicates HSPs as candidates for this role, based on these preliminary results and the mechanism of their action. In this context, a group of researchers investigated the difference in circulating HSP70 between patients with hypertensive disorders of pregnancy and pre‐eclampsia and normal controls concluded that circulating HSP70 was found to be higher in the patient group in comparison with the control group. There was, however, no observed difference between severe and mild pre‐eclampsia, early and late onset disease and between pregnancies affected or not affected by FGR.^54^ In another case–control study by the same research group,^55^ including patients with HELLP syndrome, severely pre‐eclamptic patients without HELLP syndrome and normotensive control pregnancies, HSP70 and C‐reactive protein (CRP) levels were significantly higher while alpha(2)‐HS glycoprotein (AHSG) levels were lower in the patient group.

In the same context, Madách et al.,^56^ in a case series comprising patients with HELLP syndrome, found HSP70 levels were correlated with hemolysis and hepatocellular damage parameters. However, no correlation was observed with inflammation, coagulation and renal function markers. Furthermore, a statistically significant negative correlation was observed between HSP and platelet levels.^45^, ^46^ Hromadnikova et al.^57^ used a different approach and measured the circulating mRNA levels in patients with gestational hypertension, pre‐eclampsia, FGR and women with uncomplicated pregnancies and HSP70 mRNA levels were found to be higher in the pre‐eclampsia group; however, no difference was observed between pregnancies with and without FGR.

A systematic review and pooled analysis of the current literature in 2018,^58^ including 350 pre‐eclamptic patients and 429 controls, concluded that HSP70 concentration was statistically significantly higher in pre‐eclamptic patients compared to the control group. This study highlights the potential role of HSP70 as a diagnostic and screening tool. However, before the wide use of HSPs as screening tools more studies including larger scale analysis including and other types of HSPs should be designed. Moreover, the correlation between intra‐ and extracellular (circulating) levels of HSPs should be investigated in future studies.

## 8 HEAT SHOCK AND MAMMALIAN REPRODUCTION

Global warming is recognized as a major threat for agriculture worldwide. European cattle are known to be affected by high temperatures during warm time periods. Fertility and milk production are reduced during summer, and it is thought that these changes are the result of complex physiological mechanisms involving the endocrine, immune, reproductive system and the metabolism.^59^ In a recent study it was found that supplementing the culture media of cattle zygotes with HSP70 led to the amelioration of the effect of heat shock on the formation of blastocysts.^60^ Although the data regarding humans are scarce, analogous pathways could also exist in human reproduction and should be further studied since global warming is a phenomenon that affects all.

As mammals serve as excellent models for understanding human reproductive biology, understanding the protective role of HSPs in heat shock environment in mammals is expected to promote knowledge on the effect of climate change in human reproduction. Research on heat shock and its effects on mammalian reproduction can provide critical insights into how heat stress impacts fertility, gamete quality, embryonic development, and reproductive success in humans.

## 9 FUTURE PERSPECTIVES

**FIGURE 1:**
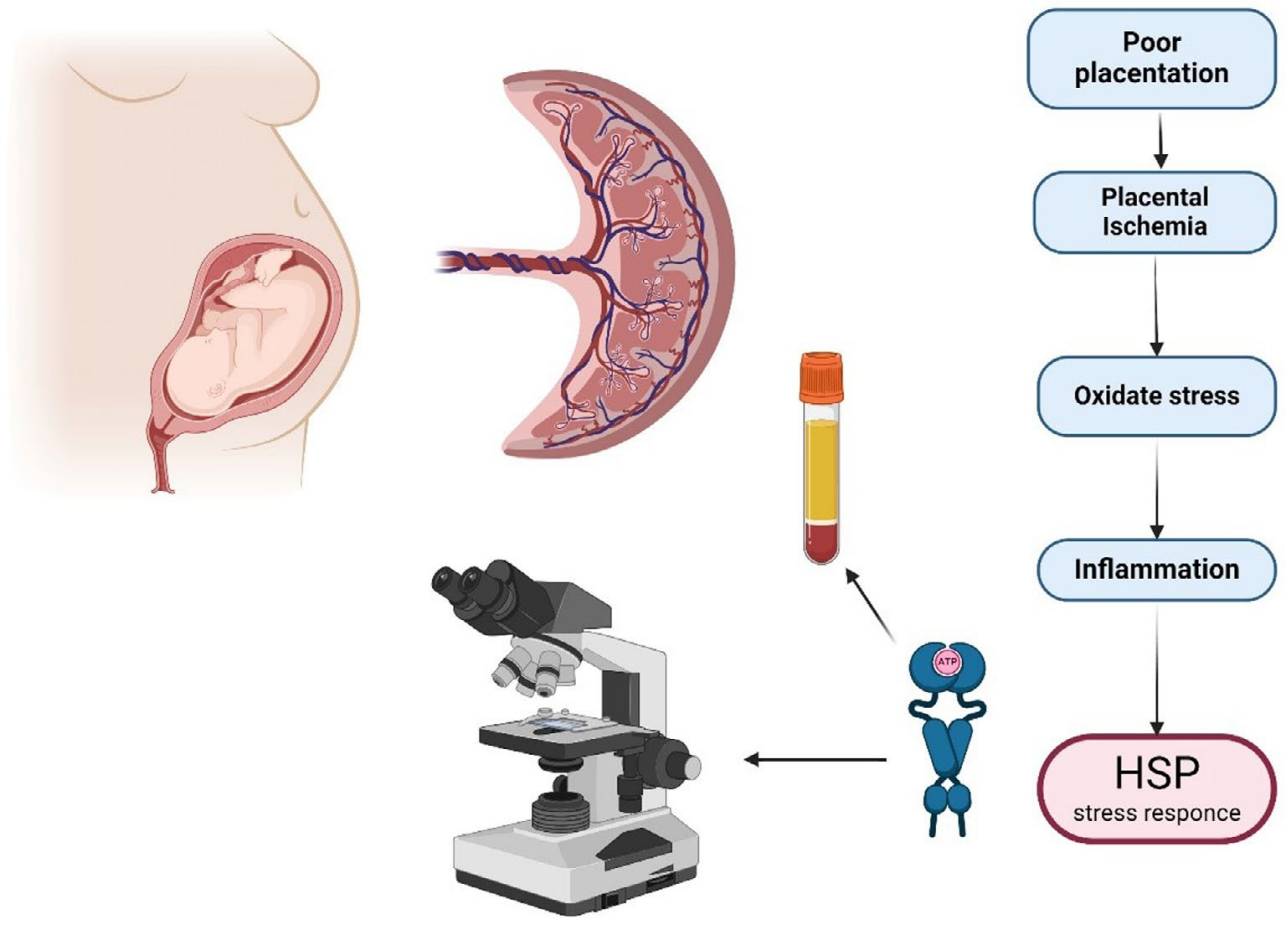
Expression of heat shock proteins (HSP) as a protective molecule in response to stress due to placental ischemia. HSP can be detected via immunochemistry and circulating mRNA.

HSPs appear to be promising biomarkers that could be used for screening and monitoring the cellular stress of the placenta and its dysfunction, a factor that is known to contribute to both FGR and pre‐eclampsia (Figure 1). Yet, in order to be able to draw a more final conclusion regarding their utility in clinical practice, there is still a long way to go. One important feature is the fact that most of the studies examining the role of HSPs have really small sample sizes. Therefore, there needs to be large scale studies so that we can obtain conclusions with an acceptable degree of statistical significance. The different methodologies used by different research groups can also be a source of confusion. That is why a commonly accepted methodology needs to be applied.

As mentioned above, to this day there are no biomarkers that can be utilized in order to monitor the response to the therapy that is being implemented. This could be a very fruitful field of research and the discovery of such biomarkers could help us standardize a more targeted therapeutical and monitoring practice. Biomarkers such as HSPs could prove to be useful especially in cases where the current sonographic criteria fail to correctly categorize pregnancies at risk of complications due to placental dysfunction. The integration of new knowledge is critical to the development of algorithms that have a purpose to individualize the approach of a patient based on their particular needs. Moreover, further studies are needed in order to investigate the impact of temperature and heat shock on human reproduction and try and precisely understand the role of HSPs in human physiology.

Finally, translating the fundamental knowledge of HSPs functions into clinical contexts requires the exploration of pharmacologic agents that can modulate the expression of HSP. The study of small molecules, such as HSP activators or inhibitors, can provide innovative paths for therapeutic intervention in case of PID.

## 10 CONCLUSION

Pre‐eclampsia and FGR represent common problems for healthcare providers of perinatal care with increased associated morbidity. HSPs may prove to be of great assistance according to many studies. Data from animal studies show that HSPs have a positive impact on mammalian reproduction. It is imperative therefore to perform more careful and targeted research in order to obtain robust data and reach solid conclusions.

## AUTHOR CONTRIBUTIONS

Conceptualization: A.A.S. and S.S. Methodology: A.A.S., A.L., M.I. and S.S. Formal analysis: A.A.S. and D.M. Investigation: A.L., S.T., A.G. and E.A. Writing–original draft, A.A.S. and A.L. Writing–review and editing, M.I., S.T, A.G, E.A., A.D. and S.S. Supervision, A.D. and S.S. All authors have read and agreed to the published version of the manuscript.

## FUNDING INFORMATION

This research received no external funding.

## CONFLICT OF INTEREST STATEMENT

The authors declare no conflict of interest.

## Data Availability

Data are available upon reasonable request.
